# How can community-based (re)engagement initiatives meet the needs of ‘NEET’ young people? Findings from the theory gleaning phase of a realist evaluation in Sweden

**DOI:** 10.1186/s13104-022-06115-y

**Published:** 2022-06-28

**Authors:** Frida Jonsson, Anne C. Gotfredsen, Isabel Goicolea

**Affiliations:** 1 Department of Epidemiology and Global Health, Umeå University, Umeå, Sweden; 2 Arctic Research Centre (Arcum) at Umeå University, Umeå, Sweden

**Keywords:** Sweden, NEET, Young people, (Re)engagement, Realist evaluation, Theory gleaning

## Abstract

**Objective:**

There has been a lack of systematic and theoretically underpinned evaluations, internationally and in Sweden, of local multi-component initiatives delivered outside public employment services and formal education systems to young people who are not in employment, education or training (‘NEETs’). To bridge this knowledge gap, the objective of this study was to present findings from the theory gleaning phase of a realist evaluation aimed at assessing how Swedish community-based initiatives may work to (re)engage vulnerable ‘NEET’ young people in education or employment, under what conditions and why.

**Results:**

Based on insights gleaned and synthesised from various sources, three candidate programme theories were elicited drawing attention to the importance of community-based initiatives in Sweden adopting a ‘caring approach’, a ‘capability approach’ and a ‘collaborative approach’ to (re)engage ‘NEET’ young people in education or employment. While limited to the initial phase of theory gleaning, the study provides valuable insights into the potential functioning of (re)engagement initiatives directed towards vulnerable ‘NEETs’ in addition to increasing the transparency of a highly iterative research project.

## Introduction

The health and life situations of young people aged 15–29 years who are neither in employment, education nor training (‘NEET’) [1–3], has gained attention across Europe [4], including Sweden [5], in recent decades. As a reflection of this focus, EU member states have implemented policies to reduce the proportion of ‘NEETs’ among young people, for example, through the recently reinforced Youth Guarantee^1^ (YG) [6]. However, despite efforts made by European governments [7], there is a lack of knowledge about what works to (re)engage ‘NEET’ young people in formal studies or paid work [8]. Considering the heterogeneity of ‘NEETs’ [9] and the fact that local outreach initiatives has been largely overlooked in national YG implementations [10], this lack of evidence is especially salient for subgroups who face complex challenges in their school-to-work transitions and for whom multi-component and contextualised, rather than singular or standardised, support may be central [11–14].

The study presented in this research note intends to bridge this knowledge gap by focusing on local (re)engagement initiatives delivered outside public employment services and formal school settings to ‘NEETs’ who run the risk of long-term precariousness, for example, due to chronic or mental illness, disability, immigrant background and early school leaving [15]. Specifically, this article presents findings from the first ‘theory gleaning’ phase of a realist evaluation [16–18] aimed at assessing how community-based initiatives that combine different practices and approaches work to (re)engage vulnerable ‘NEET’ young people in education or employment, under what conditions and why. In this regard, the study builds on previous research, which has called for systematic and theoretically underpinned evaluations that recognise the local innovation of, and nuances in delivery between, multi-component initiatives directed towards particularly disadvantaged subgroups of ‘NEETs’ [8].

Conducted in the Swedish setting, the study presented here also responds to the fact that local multi-component initiatives have been implemented, but never rigorously evaluated in Sweden, despite inquiries and agencies being commissioned to analyse the situation and propose solutions to the ‘NEET’ problem [19]. Against this backdrop, and considering the COVID-19-pandemic’s added impact on the life chances and living conditions, especially of young people like vulnerable ‘NEETs’ [20], there is a need for knowledge about ways through which their (re)engagement in education and employment can be facilitated in community settings.

## Main text

This research note presents findings from the ‘theory gleaning’ phase of a realist evaluation (RE) [18] that aims to assess how community-based initiatives may work to (re)engage vulnerable ‘NEET’ young people in education or employment, under what conditions and why. While the RE steps of theory gleaning, refinement and consolidation have received attention in recent literature [16, 17], few studies have elaborated on the application of these phases to increase the transparency of their research as we do here.

### Methodology

With origins in scientific realism, and building on the definitions in Table 1, RE rests on the premise that outcomes of complex initiatives are caused by interactions between contextual factors and underpinning mechanisms of change [21, 22]. Drawing upon data from multiple sources and retroductive theorising, whereby the researchers seek to uncover latent generative mechanisms that are responsible for the empirical manifestation of outcomes, RE have been considered suitable to provide solutions of relevance for policy and practice [23].

**Table 1:** Definitions of key realist concepts

Context (C)	Dynamic conditions that occur before, or exist outside of, the initiative with the potential to activate mechanisms during implementation [24]
Mechanisms of change (M)	Hidden or latent (i.e. real, but most likely not directly visible) aspects with the power to generate outcomes in a given context. Pertains to the reasoning and reactions of people in *response* to *resources* offered by the initiative [25, 26]
Outcomes (O)	Intended or unintended effects of the initiative with behavioural or system changes occurring at intermediate or long-term levels through interactions between context and mechanisms [22]

By being realist informed, this methodology is also theory driven [27]. This means that RE take the notion that complex initiatives are underpinned in design and implementation by implicit or explicit assumptions about how change might occur, as a point of departure. Following a cyclic, albeit iterative, structure [16, 17], the goal of RE is first to identify these assumptions and convert the theorised claims of how the initiative could or should work into *programme theories* (PTs) suitable for scrutiny. To facilitate this, information from diverse sources such as theory gleaning interviews, theory-driven literature reviews and stakeholder consultations [16] is conceptualised into context–mechanism–outcome (CMO) configurations by postulating causal links between contextual factors, mechanisms and potential outcomes [22, 23].

Once elicited, the PTs are scrutinised against data to be confirmed, refuted and revised [28]. Building on the ‘I’ll show you my theory, if you’ll show me yours’ approach [22], this process of theory refinement and consolidation usually involve interviews that follow a teacher–learner cycle where the interviewer and the interviewee engage in a dynamic conversation of thinking through the programme complexities [16]. The theories are thus the subject matter of interviews where the subject is there ‘to confirm or falsify and, above all, to refine’ them [29], p.299]. Through an iterative realist analysis that is contemporary with, and retrospective to, the fieldwork, a consolidated theory that is ‘abstract enough to underpin the development of a range of program types yet concrete enough to withstand testing in the details of program implementation’ [22], p.116] is then produced.

#### Procedure and participants: theory gleaning

Following the cyclic structure of RE [16, 17], we initiated the project presented here with a theory gleaning phase spanning across 1 year (2021). The focus of this initial phase was to glean and analyse information from various sources for the purpose of unearthing and configuring ideas that explain how community-based initiatives may work to (re)engage ‘NEET’ young people in education or employment, under what conditions and why.

The process of unearthing ideas that underpinned the initiative’s design and implementation comprised 14 theory gleaning interviews, conducted with 21 practitioners (managers and frontline workers) using exploratory questions, for example, about how the work was supposed to be carried out, for what purpose and what conditions might facilitate or hinder the process [16]. These interviews were digitally recorded, transcribed verbatim and analysed by coding aspects of context, mechanisms and outcomes guided by the definitions in Table 1, while identifying configurations between CM, MO and CMO components made by participants. In parallel with conducting the interviews, the first author initiated a theory-driven realist review that is ongoing, where international academic and grey literature on same topic has been analysed and synthesised. She also conducted 15 traditional qualitative interviews with ‘NEET’ young people for an adjacent project to learn more about their situations and need for support in addition to engaging in over 20 informal consultations with other stakeholders. As part of the theory gleaning, our analysis of information from the three latter sources was based on the first authors’ analytical notes.

The process of configuring ideas was based on insights gleaned from the above sources (i.e. the codes and configurations from the theory gleaning interviews and analytical notes from the review, traditional interviews and stakeholder consultations) during this first year, combined with several project meetings were we engaged in an exploratory process of retroductive analysis informed by over-coded abduction [18, 23]. This involved creative and hunch-driven interpretations about underlying mechanisms and contextual conditions that together could produce the outcome of ‘NEET’ young people (re)engaging in education or employment. Based on these discussions and through the dynamics of writing up the findings involving oscillations between, and cross-checking the creative and hunch-driven interpretations across, the codes/initial configurations and analytical notes, we developed three candidate PTs as presented below.

### Findings

Based on insights and analyses from the theory gleaning, three PTs were elicited. Below, these have been cast as *if*–*then* propositions to render them into their ‘constituent and interconnected elements’ [21], p.39].

PT 1 *‘the caring approach’*: If non-judgemental practitioners can tune into the emotional and physical world of the young people and communicate this continuously through reciprocity, respect and recognition, then this will contribute to the establishment of a caring professional relationship through which the participants’ engagement and motivation can be nurtured by making them feel valued and cared for. Specifically, rather than demanding change via artificial sensitivity or a controlling system, young people’s (re)engagement in education or employment can be facilitated if supported by a professional relationship characterised by genuine understanding, negotiation and a delicate balance between support and expectations.

PT 2 ‘*the capability approach*’: If the initiative can offer, and practitioners engage participants in, activities that align with their varying needs and aspirations while providing space for them to exercise choice, then the young people will be motivated to stay in the programme and progress by developing relevant capabilities. In addition, if the practitioners can divide the learning process into manageable steps while providing room for reflection, then the young people can have the possibility to gain a sense of achievement while accumulating a series of successes that instil pride and confidence. This, in turn, will ensure that the young people stay engaged in the process of developing capabilities and progress to (re)engage in education or employment. Conversely, constraints on the activities provided may lead to further disengagement by young people feeling bored, disappointed or disregarded unless they can be negotiated to achieve agreed alternatives through a caring professional relationship.

PT 3 ‘*the collaborative approach*’: If the initiative can coordinate activates with government agencies as well as specialist health and social services that are willing and able to work in collaboration through mutuality and accountability, then the young people will gain a sense of entitlement while accessing through clear referrals the care needed to manage urgent or serious challenges in their lives. If provided in parallel with other activities this will, in turn, ensure that they can stay engaged in the process of developing relevant capabilities to (re)engage in education or employment by overcoming issues that may otherwise have acted as barriers to their (re)engagement.

### Next steps

During the upcoming years (2022–23), the three candidate PTs will be confirmed, refuted or revised through theory refinement and consolidation to assess how community-based initiatives work to (re)engage vulnerable ‘NEET’ young people in education or employment, under what conditions and why.

#### Procedure and participants: theory refinement and consolidation

We will start by conducting, and analysing data from, theory refinement interviews while triangulating the information with reviews of programme documents and participatory observations [17]. As previously explained, the interviews will follow a teacher–learner cycle where the focus will be to discern, in dialogue with the participants, what aspects of the PTs are applicable and accurate [16]. For this purpose, and to capture a range of understandings, the PTs will be articulated in an interview guide and presented separately to managers, frontline workers and young people who should use their experiences and expertise as tools for theory refinement [22].

As the collected and analysed data from practitioners, young people, programme documents and participatory observations accumulate with comparisons being made across sources of information, we will engage with key participants again to fine-tune the theories [17]. By probing issues that require further clarification, theory consolidation interviews will be conducted with a few practitioners and young people to assess how the PTs contribute (or not) to our understanding about the functioning of community-based (re)engagement initiatives.

Ultimately, rather than aiming for consensus or saturation, the data collected and analysed in the theory refinement and consolidation phases will continue until sufficient variability to ‘move from constructions to explanation of causal mechanisms’ have been achieved [16] (p.348). Moreover, to ensure that the fieldwork can be informed by emergent findings in the process of developing a consolidated theory, an iterative and retroductive analysis will be conducted in parallel to the data collection to unearth mechanism in contexts that contribute to the outcome of (re)engaging ‘NEET’ young people in education or employment [18, 23].

## Limitations

Although we do not outline the final steps or results of our RE in this research note, presenting findings from the first theory gleaning phase while outlining the steps ahead is an important and novel contribution to the field. Firstly, because the process of eliciting PTs is seldom made explicit in RE publications, and secondly, since an evaluation of this kind has never been conducted before even candidate PTs may provide information useful for programme developers and implementers.

Regarding the upcoming process of data collection and analysis, when presented with the PTs it is possible that participants may simply agree with our propositions, a phenomenon known as acquiescence. However, following Mukumbang et al. [17], we will try to minimise this risk by inviting the participants to exemplify and provide insight into instances when the initiative worked (or not) according to the theory. Furthermore, in this research note we have presented three candidate PTs that provide a set of complementary propositions of how community-based (re)engagement initiatives may work. In the process of scrutinising the PTs through theory refinement and consolidation, we will nevertheless remain open and sensitive to the possibility of finding rival or oppositional explanations to expected or unexpected outcomes if the evidence points us there.

## Data Availability

The data analysed is not publicly available because it contain sensitive information but may be available from the corresponding author on reasonable request.

## Abbreviations

NEET: Not in employment, education, or training
CMO: Context–mechanism–outcome
PTs: Programme theories
RE: Realist evaluation

## Footnotes

1 The reinforced Youth Guarantee represented a continued commitment by all EU Member States to ensure that all young people under the age of 30 receive a good quality offer of employment, continued education apprenticeship, traineeship within a period of four months of becoming unemployed or leaving education.
